# Orphan Genes Find a Home: Interspecific Competition and Gene Network Evolution

**DOI:** 10.1371/journal.pgen.1005254

**Published:** 2015-06-18

**Authors:** Karley K. Mahalak, Helen M. Chamberlin

**Affiliations:** 1 Department of Molecular Genetics, Ohio State University, Columbus, Ohio, United States of America; 2 Graduate Program in Molecular, Cellular and Developmental Biology, Ohio State University, Columbus, Ohio, United States of America; Stanford University Medical Center, UNITED STATES

Conservation of gene and protein sequence, and, therefore, conservation of the resulting molecular interactions that mediate biological processes, is foundational to our understanding of biology. This conservation allows discovery in one organism, such as worms or mice, to inform our understanding of the biology in another organism, such as humans. However, there is emerging recognition that many biological processes involve important non-conserved elements, and that de novo gene birth provides an important mechanism for functional evolution [1–3]. Understanding how these novel elements incorporate into gene regulatory networks and alter the network architecture is an important area for theoretical research [4–5], but few experimental examples have been described.

Formation of the dauer larva by nematodes is an adaptation that is responsive to a variety of environmental cues and alterations, features that suggest the underlying gene regulatory network might benefit from enhanced robustness and evolvability [6]. The dauer larva is a relatively dormant, alternative developmental stage that nematodes enter under stressful conditions, such as low food or crowding (signaled by pheromone), that confers increased longevity and tolerance to stressors [7]. In many parasitic nematodes, this stage corresponds to the infective larval stage when the larvae transition to new hosts. There are important conserved components of the gene network that regulate dauer formation, one being the nuclear hormone receptor DAF-12, which is important in free-living as well as parasitic nematode species [8–10]. However, it is also clear that this pathway is subject to considerable inter- and intraspecific differences [11–13]. Recent work on natural isolates of the nematode *Pristionchus pacificus* identified strain-specific phenotypic differences in dauer formation, and argued that genetically distinct populations exhibit greater sensitivity to pheromone from other populations (pheromone cross-preference [14]). The genetic alterations responsible for these phenotypic differences, however, had not yet been identified.

**Fig 1:**
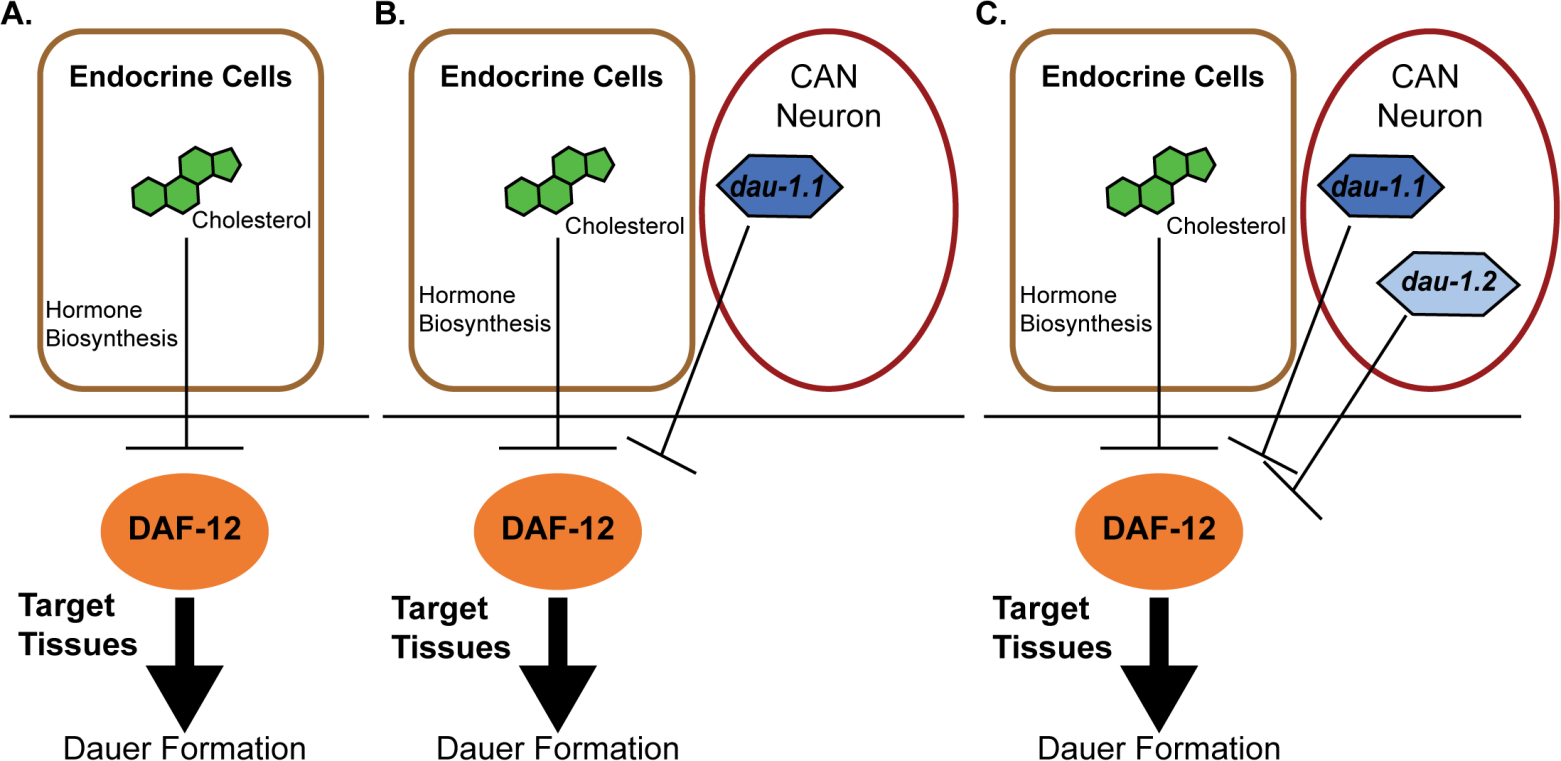
Proposed model for genetic evolution in *P*. *pacificus* dauer formation. A) Conserved features of the dauer formation pathway. In endocrine cells, cholesterol is a substrate for the biosynthesis of dafachronic acid (DA), a DAF-12 ligand that suppresses the ability of DAF-12 to promote dauer formation. B) Model for the RS5134/Ohio strain with one copy of *dauerless (dau-1*.*1)*. *dau-1*.*1* is expressed in the CAN neuron cells and acts (genetically) to inhibit DAF-12 function and thereby inhibit dauer formation. C) The RS2333/California strain has two copies of *dauerless* (*dau-1*.*1* and *dau-1*.*2*), and a corresponding double effect of inhibition of dauer formation.

A new paper in this issue of *PLOS Genetics* [15] provides an answer, and at the same time highlights how “orphan” genes can be incorporated into conserved regulatory networks. The authors started with two strains of *P*. *pacificus* that exhibited marked differences in response to pheromone signaling: the RS2333/California strain, with low dauer formation in response to pheromone, and the RS5134/Ohio strain, with a high response. They then generated 911 recombinant inbred lines (RIL) between the strains, and used Quantitative-Trait-Loci (QTL) mapping to identify a novel gene with no apparent orthologs outside of Pristionchus, *dauerless* (*dau-1*), that had undergone a duplication event in the RS2333/California strain. This suggested that *dau-1* functions as a repressor of dauer formation in a dose-dependent manner. To test this hypothesis, the authors created transgenic lines with multiple copies of *dau-1*, and created deletion mutants using CRISPR/Cas9 technology. Their data support the hypothesis as *P*. *pacificus* animals with more than two gene copies show no dauer formation in response to pheromone, whereas the deletion of one or both gene copies in RS2333 results in significantly higher dauer formation. The authors also show that this function is mediated by the CAN neurons. *dau-1* is expressed in these cells, and ablation of these neurons results in increased dauer formation similar to that seen in *dau-1* mutants. Finally, epistasis experiments show that *dau-1* acts either downstream or in parallel to steroid hormone signaling, but is dependent on the nuclear hormone receptor gene *daf-12* (Fig 1).

Discovery of the *dau-1* genes provides insight for how orphan genes can play an important role in the function and the evolution of biological networks that are conserved across species. The presence—and the duplication—of this gene is interpreted to provide a selective advantage in the context of intraspecific competition because it would allow individuals to continue with a reproductive life cycle even in the presence of crowding. Indeed, Mayer et al. have uncovered additional *dau-1* paralogs in RS2333, indicating a potential for ongoing duplication of these novel genes [15]. A second important finding of this paper is that these orphan genes influence dauer formation by modulating a pathway with highly conserved elements (Fig 1). The results provide an important experimental example to complement the theoretical models for how the evolution of novel genes can add functional modifications to conserved regulatory networks. In this case, orphan gene evolution contributes to the evolutionary arms race between competing strains.

Important questions remain. In particular, previous work showed that different *P*. *pacificus* isolates exhibit differences in pheromone signaling and dauer survival, in addition to pheromone response, arguing that there are additional genetic modifications in the dauer regulatory network [14]. Whether these functions are influenced by *dau-1* or related genes, and how these features of the dauer regulation network may interact, is not known. In addition, whether this represents a unique example or an evolutionary prototype is not clear. Generalizations of the types of changes that are responsible for evolutionary change have focused on the level of individual orthologous (and, therefore, conserved) genes [16–17]. These earlier analyses did, however, highlight how the network position of a gene can influence whether it is likely subject to evolutionary modification, and the types of network nodes that are more likely to be affected by cis-regulatory or protein-coding changes. As more evolutionary examples involving orphan genes are described, it will be interesting to learn if they are preferentially incorporated into particular nodes of regulatory networks, and whether they contribute primarily to intraspecific differences or have a role at longer evolutionary timescales.
